# An algorithm based on the postoperative decrease of albumin (ΔAlb) to anticipate complications after liver surgery

**DOI:** 10.1186/s13741-022-00285-w

**Published:** 2022-11-09

**Authors:** Ismail Labgaa, Luis Cano, Orsalia Mangana, Gaëtan-Romain Joliat, Emmanuel Melloul, Nermin Halkic, Markus Schäfer, Eric Vibert, Nicolas Demartines, Nicolas Golse, Martin Hübner

**Affiliations:** 1 Department of Visceral Surgery, Lausanne University Hospital CHUV, University of Lausanne (UNIL), Rue du Bugnon 46, CH-1011 Lausanne, Switzerland; 2 Nutrition Metabolism and Cancer, INSERM, University of Rennes, INRAE, CHU Pontchaillou, UMR 1241 NUMECAN, Rennes, France; 3 Department of Hepatobiliary Surgery, Paul-Brousse Hospital, Assistance Publique Hôpitaux de Paris, Centre Hépato-Biliaire, 94800 Villejuif, France; 4 Université Paris-Saclay, Inserm, Physiopathogénèse et traitement des maladies du Foie, UMR-S 1193, Paris, France

**Keywords:** Partial hepatectomy, Liver resection, Decision-making, Morbidity, Biomarkers, Predictors

## Abstract

**Background:**

Perioperative decrease of albumin (ΔAlb) appeared as a promising predictor of complications after digestive surgery, but its role after liver surgery remains unclear. This study aimed to analyze whether and how ΔAlb can be used to predict complications after liver surgery.

**Methods:**

A bicentric retrospective analysis of patients undergoing liver surgery (2010–2016) was performed, following TRIPOD guidelines. The preoperative and postoperative difference of albumin was calculated on POD 0 and defined as ΔAlb. Patients with any missing variable were excluded. The primary endpoint was overall complications according to the Clavien classification. A multiparametric algorithm based on ΔAlb was generated to optimize prediction performance.

**Results:**

A total of 110 patients were analyzed. At least one complication occurred in 66 (60%) patients. Patients with and without complication showed a ΔAlb of 15.8 vs. 9.5 g/L (*p*<0.001). Area under ROC curve (AUC) of ΔAlb was 0.75 (*p*<0.01.). The ΔAlb-based algorithm showed an AUC of 0.84 (*p*<0.01), significantly improving performance (*p*=0.03). Multivariable analysis identified ΔAlb as independent predictor of complications (HR, 1.12; 95% CI, 1.01–1.07; *p* = 0.002).

**Conclusions:**

ΔAlb appeared as a promising predictor independently associated with the risk of complication after liver surgery. The study presents a novel decision-tree based on ΔAlb to anticipate complications.

**Supplementary Information:**

The online version contains supplementary material available at 10.1186/s13741-022-00285-w.

## Background

Liver surgery entails major surgical procedures that trigger intensive physiological stress. Mortality and morbidity may reach 10% and 45%, respectively (Jarnagin et al. 2002; Vonlanthen et al. 2011; Breitenstein et al. 2010; Labgaa et al. 2019; Filmann et al. 2019). Reducing the incidence of adverse outcomes is challenging, and early detection is paramount. Identification of reliable tools capable to predict and anticipate postoperative complications is the first step. Ideal candidate predictors must be performant, easy to measure and inexpensive, but also importantly, must be early indicative (Labgaa et al. 2017a, b; Longchamp et al. 2021).

The perioperative variation of albumin (ΔAlb) is a novel marker that showed promising results in digestive surgery (Labgaa et al. 2016a, 2017c, 2021; Muller et al. 2018). Unlike preoperative single values of serum albumin, it has not been extensively investigated. To date, less than 20 studies explored ΔAlb in abdominal surgery (Joliat et al. 2022). In major abdominal surgery, ΔAlb ≥10 g/L was identified as independent predictor of complications (Labgaa et al. 2017c). Müller et al. showed that ΔAlb was better prognostic marker than absolute albumin value in a cohort of patients undergoing intestinal resection for Crohn’s disease (Muller et al. 2018). Nonetheless, the potential role of ΔAlb to predict complications after liver surgery remains unknown. Aside orthotopic liver transplant (OLT), data on ΔAlb remain particularly scarce for liver surgery patients. In 2016, a pilot study from our group showed that ΔAlb was an independent predictor of complication (Labgaa et al. 2016a). However, this study did not provide a specific cutoff. These results needed to be confirmed with a larger sample size and an external dataset, allowing to also establish a threshold for ΔAlb.

The present study aimed to assess the predictive value of ΔAlb for adverse outcomes and to define how it may help anticipating postoperative complications in patients undergoing liver surgery.

## Methods

### Design

This bicentric retrospective study was conducted in the departments of surgery at Lausanne University Hospital (CHUV) and at Paul-Brousse Hospital in Paris, between 2010 and 2016, following TRIPOD guidelines. The study was conducted in accordance with the Declaration of Helsinki, and it conformed to good clinical practice guidelines. Informed consent was obtained from all included patients. The protocol was approved by the Institutional Review Boards at University Hospital of Lausanne (CER-VD # 2017-01169) and at Paul-Brousse Hospital (CO 16-006).

### Patients

Consecutive patients >18 years undergoing liver surgery during the study period were included. Very stringent selection criteria were applied: patients with any missing variable were excluded. Collected data included demographics, surgical, and perioperative variables as well as postoperative outcomes. Data were retrieved from institutional databases.

### ΔAlb

Blood samples were collected by nurses preoperatively and postoperatively, allowing to obtain daily measures of serum albumin concentration [g/L]. Perioperative variation of albumin was defined as ΔAlb and calculated according to the following formula: preoperative albumin value - albumin value on POD 0 (4–8 h after the end of surgery). Timepoints were selected to tailor a predictor being indicative as early as possible (POD 0), as well as based on data from a previous pilot study (Labgaa et al. 2016a).

### Endpoints

Postoperative morbidity was graded according to the Clavien classification (Dindo et al. 2004). Minor and major complications were defined as grades I–II and III–IV, respectively. Postoperative mortality was defined as grade V. The primary endpoint was overall morbidity while secondary endpoints were Comprehensive Complication Index (CCI) (Slankamenac et al. 2013) and postoperative length of stay (LoS) (Labgaa et al. 2016b; Joliat et al. 2016).

### Statistical analyses

All statistical analyses were conducted by a statistician (L.C.). Complete case study technique and bootstrap methods (1000 repetitions) were conducted to handle missing data. Single imputation method was avoided due to the risk of bias in small populations (Dziura et al. 2013; Zhang et al. 2017; Jakobsen et al. 2017).

Continuous variables were provided as median with interquartile range (IQR) or as mean with standard deviations (SD), according to their distribution pattern. They were compared with Student’s *t* test or Mann-Whitney *U* test. Categorical data were provided as frequencies with percentages and compared with either *χ*^2^ or Fisher’s exact test. Receiver operating curve was generated, allowing to calculate area under the curve (AUC). Uni- and multi-variable analyses were performed. Variables with *p*<0.1 on univariable analyses were further considered in the multivariable analysis, integrating multiple potential confounding factors into the model. To increase the accuracy of variables selection, a stepwise regression in both senses was used (forward and backward). The best model was selected based on AUC values. A decision-tree was generated to optimize prediction performance. Its number of nodes were minimized on purpose, to facilitate clinical implementation. Hence, the model integrated the strongest predictors identified on univariate analyses (*p*<0.001). A bootstrap technique with 1000 repetitions was conducted to overcome the relatively small sample size. Standard error and cross-validation error values were analyzed, in order to determine the optimal cutoff points, preserving as much accuracy as possible. DeLong test was used to compare AUC curves. The statistical significance was defined as a *p* value <0.05. The statistical analyses were performed with the statistical software R 4.0.1 (Core Team 2020), and the following packages were used to develop the statistical analysis: decision trees: part (4.1–15, Terry Therneau and Beth Atkinson (2019)), roc curves: ROCR (1.0–11, Sing T, Sander O, Beerenwinkel N, Lengauer T (2005)), data manipulation: dplyr (0.8.5, Hadley Wickham, Romain François), and statistical modeling: caret (6.0.86, Max Kuhn (2020)).

## Results

### Patients and outcomes

After exclusion of patients with any missing variable, a total of 110 patients were included in the final analyses. Patients’ characteristics and details of surgery are provided in Table 1. Regarding outcomes: minor, major, and overall complications were reported in 59 (53.6 %), 22 (20 %), and 66 (60 %) patients, respectively. Postoperative mortality was reported in 1 patient (0.9%). Median CCI was 19.14 (0–100) while median LoS was 14.3 days (3–67). Characteristics of the final cohort were compared with the excluded patients in Supplementary Table 1.

**Table 1:** Patients characteristics and details of surgery

	Value Mean (±SD) or ***n*** (%)
Age (years)	61.37 (10.48)
Gender (female)	39 (35.5 %)
Smoking	30 (27.7 %)
Diabetes	87 (79.1 %)
Cirrhosis	9 (8.2 %)
Cancer	85 (77.3 %)
HCC	12 (10.9 %)
CCa	14 (12.7 %)
CRLM	46 (41.8 %)
Other malignancy	13 (11.8 %)
Non-cancer	25 (22.7%)
Alveolar echinococcosis	10 (9.1%)
Cystic echinococcosis	2 (1.8%)
Adenoma	2 (1.8%)
Hemangioma	2 (1.8%)
Others	9 (8.2%)
BMI (kg/m^2^)	26.51 (4.18)
Major resection (≥3 segments)	52 (47.3 %)
Laparoscopic approach	16 (14.5 %)
Surgery duration (min)	324.55 (132.55)
Blood loss (mL)	985 (956)
Complications (Clavien classification)	
Grade I	28 (25.5 %)
Grade II	48 (43.6 %)
Grade IIIa	15 (13.6 %)
Grade IIIb	10 (9.1 %)
Grade IVa	4 (3.6 %)
Grade IVb	3 (2.7 %)
Grade V	1 (0.9 %)

*Abbreviations*: *BMI* body mass index, *HCC* hepatocellular carcinoma, *CCa* cholangiocarcinoma, *CRLM* colorectal liver metastases

### Perioperative profile of albumin and ΔAlb

**Fig. 1:**
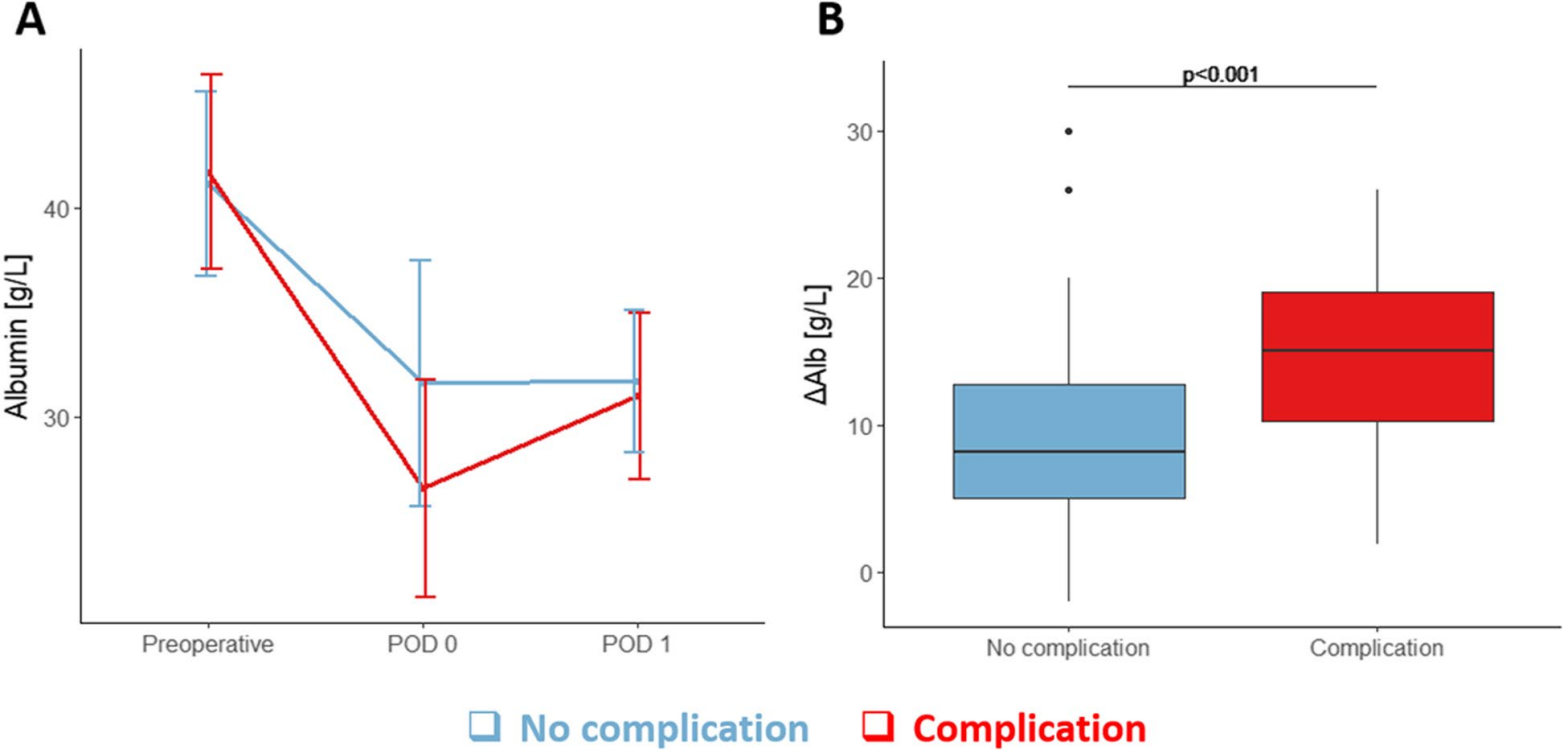
Perioperative profile of albumin and ∆Alb. **A** Perioperative profile of albumin: curves reflect median values of albumin [g/L] and bars represent interquartile range. **B** ∆Alb: boxplots of perioperative albumin variation (∆Alb). Blue illustrates patients with no complication whereas red indicates patients with any complication

Liver surgery induced a sudden decrease of albumin on POD 0, followed by a plateau phase (Fig. 1A). Although showing a similar pattern, patients who developed complication (of any grade) showed more accentuated albumin drop, compared to those without complication. Median ΔAlb was 9.5 g/L (2–30) without and 15.8 g/L (1.9–26) with complications (*p*<0.001) (Fig. 1B). For the whole cohort, median ΔAlb was 12.5 g/L (2–30). The correlation analysis was conducted to investigate the impact of intraoperative fluid perfusion on ΔAlb, showing a low correlation coefficient (Pearson=0.341, *p*=0.001), suggesting that the volume of fluid has a marginal effect on albumin drop (Supplementary Fig. 1).

### AUC, cutoff of ΔAlb, and its ability to discriminate adverse outcomes

**Fig. 2:**
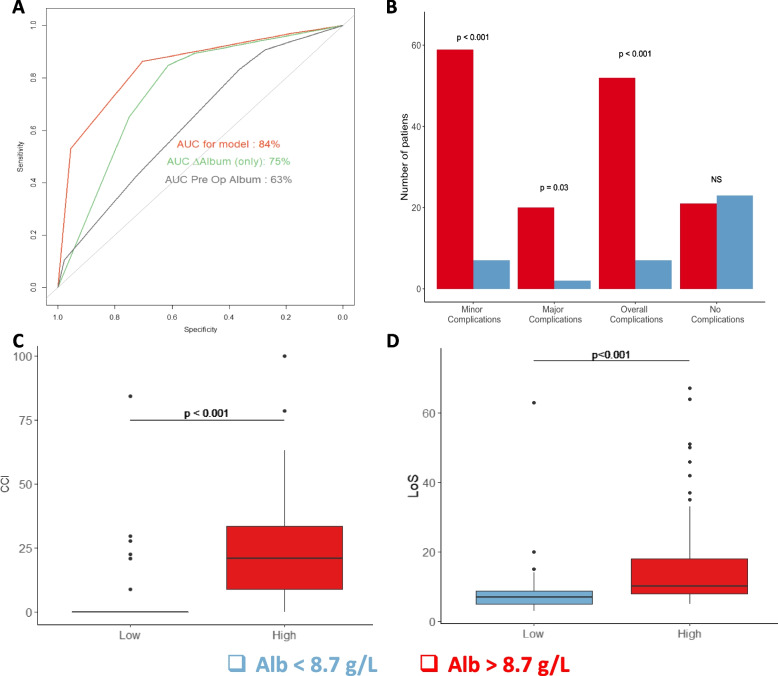
∆Alb cutoff and its discriminative performance. **A** Receiver operating characteristic (ROC) curves of preoperative albumin (gray), ∆Alb (green), and of the algorithm (orange) for overall complications of ∆Alb (blue). Calculated area under the curve (AUC) of preoperative albumin, ∆Alb, and of the model were 0.63 (*p*<0.01), 0.75 (*p*<0.01), and 0.84 (*p*<0.01), respectively. **B** Postoperative complications: bars indicating the number of patients with of minor-, major-, and overall complications. **C** Boxplots for comparison of Comprehensive Complication Index (CCI). **D** Boxplots for comparison of length of stay (LoS)

ROC curves of preoperative albumin and ΔAlb showed an AUC of 0.63 (*p*<0.01.) and 0.75 (*p*<0.01), respectively (Fig. 2A); ΔAlb curve revealed a threshold of 8.7 g/L. This yielded a sensitivity of 61.4%, specificity of 84.9%, positive predictive value of 73 %, and negative predictive value of 76.7%. It was tested whether this cutoff could discriminate patients with adverse outcomes. Minor, major, and overall complications were more prevalent in patients with ΔAlb ≥8.7 g/L (Fig. 2B). These patients also showed increased CCI of 20.90 compared to 7.53, *p*<0.001 (Fig. 2C). Likewise, LoS was also prolonged in this group of patients (16.1 *vs.* 7 days, *p*<0.001) (Fig. 2D).

### Multivariable analysis

Univariable and multivariable analyses were performed to determine whether ΔAlb was an independent predictor of overall postoperative complications (Table 2). ΔAlb (OR, 1.12; 95% CI, 1.01–1.07; *p* = 0.002) and surgery duration (OR, 1.00; 95% CI, 1.00–1.00; *p* = 0.03) were the only factors showing a statistical significance on multivariable analysis. Preoperative value of albumin itself was not predictive in univariable analysis.

**Table 2:** Multivariable analysis for overall complications

	Univariable			Multivariable		
	HR	95% CI	***p*** value	HR	95% CI	***p*** value
Age (years)	1.00	0.98–1.01	0.83			
Gender (female)	0.57	0.49–1.07	0.16			
BMI (kg/m^2^)	1.05	0.98–1.05	0.28			
Smoking	1.21	0.72–1.37	0.66			
Diabetes	4.04	1.12–1.59	0.01	2.63	0.88-1.57	0.14
Cirrhosis	3.28	0.6–1.48	0.98			
Cancer	0.64	0.49–1.13	0.35			
Major resection	1.39	0.80–1.41	0.39			
Surgical approach (open)	4.06	1.22–2.76	0.01	1.25	0.41-2.26	0.74
Surgery duration (min)	1.00	1.00–1.01	<0.001	1.00	1.00-1.00	0.03
Blood loss (mL)	1.00	1.00–1.00	<0.001	1.00	0.99-1.00	0.1
Preoperative albumin (g/L)	1.02	0.97–1.04	0.5			
∆Alb (POD 0)	1.16	1.03–1.08	<0.001	1.12	1.01-1.07	0.002

Values are presented with two decimals

*Abbreviations*: *BMI* body mass index, *CI* confidence interval, *OR* odds ratio, *POD* postoperative days

### An algorithm based on ΔAlb to optimize the predictive performance

**Fig. 3:**
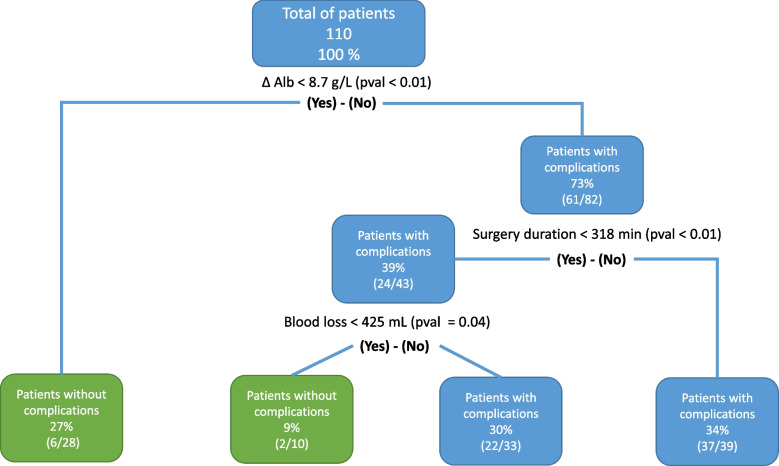
A decision-tree based on ∆Alb to optimize prediction performance. Decision-tree integrating ∆Alb, surgery duration, and blood loss to predict overall complications. Green boxes indicate group of patients at lower risk

A decision-tree was generated based on the results of the univariable analyses to improve the predictive performance. The model was designed to be easy to use on purpose and to facilitate clinical implementation (Fig. 3). It integrates ΔAlb, surgery duration, and blood loss. Its performance showed an AUC of 0.84 (*p*<0.01) (Fig. 2A), yielding a sensitivity of 70.5 %, a specificity of 86.4 %, a positive predictive value of 77.5 %, and a negative predictive value of 81.4 %. Delong test revealed a statistically increased performance of this model compared to ΔAlb alone (*p*=0.03).

## Discussion

This study adds novel data on the promising role of ΔAlb and support its use to anticipate adverse outcomes in liver surgery patients. These findings evidenced that ΔAlb was independently associated with the risk of complications and provide an easy-to-use decision-tree to predict complications after liver surgery.

As expected, liver surgery induced an abrupt fall of albumin, already on POD 0. This drop was followed by a plateau and recovery phases, which was consistent with a pilot study in liver surgery, as well as in other types of surgery (Labgaa et al. 2016a, 2017c; Ryan et al. 2007; Hubner et al. 2016). Questions are (I) what determines the amplitude of this decrease and (II) what is its clinical relevance. Regarding albumin drop, previous studies in clinical research have tried to investigate its determinants and mechanisms. Capillary leakage of albumin from plasma to tissues seemed to be a major cause (Amouzandeh et al. 2018). Dedicated investigations in basic/translational research are however needed to thoroughly decipher the precise underlying mechanisms of albumin decrease after surgery; such data as well as animal models are unfortunately not available yet. The present study was not designed to explore those elements and cannot speculate on candidate mechanisms. The amplitude of albumin decrease seems to be clinically pertinent based on its capacity to discriminate patients who will develop adverse outcomes and independent predictive value for postoperative complications. Not only novel, the clinical impact of these results is also emphasized by the fact that ΔAlb displays all the characteristics of ideal predictor: very early indicative (POD 0), easy to measure, and inexpensive. Of note, ΔAlb was superior to preoperative value of albumin, as demonstrated by AUC and multivariable analyses; the latter showed a poor performance with an AUC of 0.63 (*p*<0.01). This is consistent with other data in colorectal surgery (Muller et al. 2018).

Similar studies have highlighted the rationale to measure perioperative albumin fluctuation in upper-GI (Ryan et al. 2007), colorectal (Muller et al. 2018; Wierdak et al. 2018), and thoracic surgery (Li et al. 2018). Beside a pilot study from our group raising the interest to monitor albumin in patients undergoing partial hepatectomy (Labgaa et al. 2016a), albumin has been more investigated in OLT. Amouzandeh et al. used OLT as a model to study albumin kinetics after major surgery, allowing to identify capillary leak as a major source of albumin decrease (Amouzandeh et al. 2018). Exploring ΔAlb impact on clinical outcomes after OLT, Hiroi et al. detected an association with ΔAlb and the need for hemodynamic support (Hiroi et al. 2019). In living donors for OLT, analyses revealed that high ΔAlb was associated with an increased risk of pleural effusion (Jeong et al. 2019). Albeit being of indisputable interest, most of these studies calculated ΔAlb on POD2. In the present study, ΔAlb was calculated on POD 0, only 4–8 h after surgery, a striking advantage.

Limitation of this study is mainly its retrospective design. However, it is a multicenter effort, involving two tertiary centers. The relatively small sample size may also be considered as a shortcoming. However, selection was very stringent to obtain very clean data and to minimize the risk of bias. An important question is to know whether this stringent selection could have induced a bias. As showed in Supplementary Table 1, included patients only showed statistically higher values of BMI and blood loss compared to the excluded ones. Another limitation was related to albumin perfusion. Unfortunately, these data were not available. Future studies are needed to investigate whether and how perioperative albumin perfusion may mitigate ΔAlb and improve outcomes. Dedicated studies are needed to clarify the value of albumin perfusion intra-operatively as well as postoperatively. The model displays a descent performance that might be however overfitted to this bicentric cohort. Therefore, validating this model in a future study with an independent cohort would be important. Although it would have been valuable to validate these findings in an independent cohort, one must note that the vast majority of comparable studies investigating biomarkers lacks validation cohort, upfront. Comparison with other markers can be argued but was not an objective of the present study. ΔAlb has already been compared to other lab tests such as lactates, C-reative protein (CRP), and procalcitonin in previous studies demonstrating a higher performance (Longchamp et al. 2021; Labgaa et al. 2016a).

In addition, the present study provided a decision-tree which is easy to use and to implement in clinical practice, offering a “precision medicine” perspective. This tool may help to tailor postoperative surveillance on an individual patient basis. As example of potential clinical application, it may help guiding decision whether to transfer patients to intensive care unit (ICU), intermediate care, or to the ward. Recent data from our group also highlighted the interest of following ERAS guidelines for liver surgery, showing a significantly lower ΔAlb in patients undergoing liver surgery with ERAS, compared to non-ERAS management (Gonvers et al. 2021). Other important potential application would be to select patients who should benefit from specific therapeutic measures which would have to be tested with randomized clinical trials.

## Conclusions

These results showed an association between perioperative albumin fluctuation (ΔAlb) and the risk of postoperative complication after liver surgery, evidencing the role of ΔAlb as a predictor of adverse outcomes and the rationale to monitor it in liver surgery patients. This study also presents a novel algorithm based on ΔAlb optimizing prediction performance and helping for decision-making during the postoperative phase.

## Supplementary Information

**Additional file 1: Supplementary Table 1.** Characteristics of the final cohort were compared with the excluded patients.

**Additional file 2: Supplementary Figure 1.** Intraoperative fluid [mL].

## Data Availability

The datasets used and/or analyzed during the current study are available from the corresponding author on reasonable request.
