# Supplementary material for: An algorithm based on the postoperative decrease of albumin (ΔAlb) to anticipate complications after liver surgery
Source: Perioper Med (Lond). 2022 Nov 9;11:53. doi: 10.1186/s13741-022-00285-w (PMC9647979; doi:10.1186/s13741-022-00285-w)
Supplement: Supplementary file 1 — Additional file 1: Supplementary Table 1. Characteristics of the final cohort were compared with the excluded patients. [file 13741_2022_285_MOESM1_ESM.docx]

| **Variables** | Included patients  N = 110 | Excluded patients  N = 146 | p-value |
| --- | --- | --- | --- |
| **Age** | Mean = 61.4  SD = 10.5 | Mean = 61.8  SD = 14.5 | 0.37 |
| **Gender**  Male  Female | 71  39 | 82  63 | 0.24 |
| **Smoking**  Yes  No | 30  80 | 50  95 | 0.27 |
| **Diabetes**  Yes  No | 87  23 | 112  32 | 0.92 |
| **Cirrhosis**  Yes  No | 101  9 | 124  19 | 0.27 |
| **Cancer (previous history)**  Yes  No | 25  85 | 32  114 | 0.99 |
| **BMI (kg/m^2^)** | Mean = 26.5  SD = 4.18 | Mean = 25.7  SD = 5.31 | 0.03 |
| **Major resection**  Yes  No | 52  58 | 74  71 | 0.63 |
| **Surgical approach (Laparoscopy)**  Yes  No | 16  94 | 30  113 | 0.24 |
| **Surgery duration (min)** | Mean = 311  SD = 121 | Mean = 330  SD = 126 | 0.22 |
| **Blood loss (mL)** | Mean = 987  SD = 956 | Mean = 736  SD = 713 | 0.02 |
