# Supplementary figures and images for: An algorithm based on the postoperative decrease of albumin (ΔAlb) to anticipate complications after liver surgery
Source: Perioper Med (Lond). 2022 Nov 9;11:53. doi: 10.1186/s13741-022-00285-w (PMC9647979; doi:10.1186/s13741-022-00285-w)

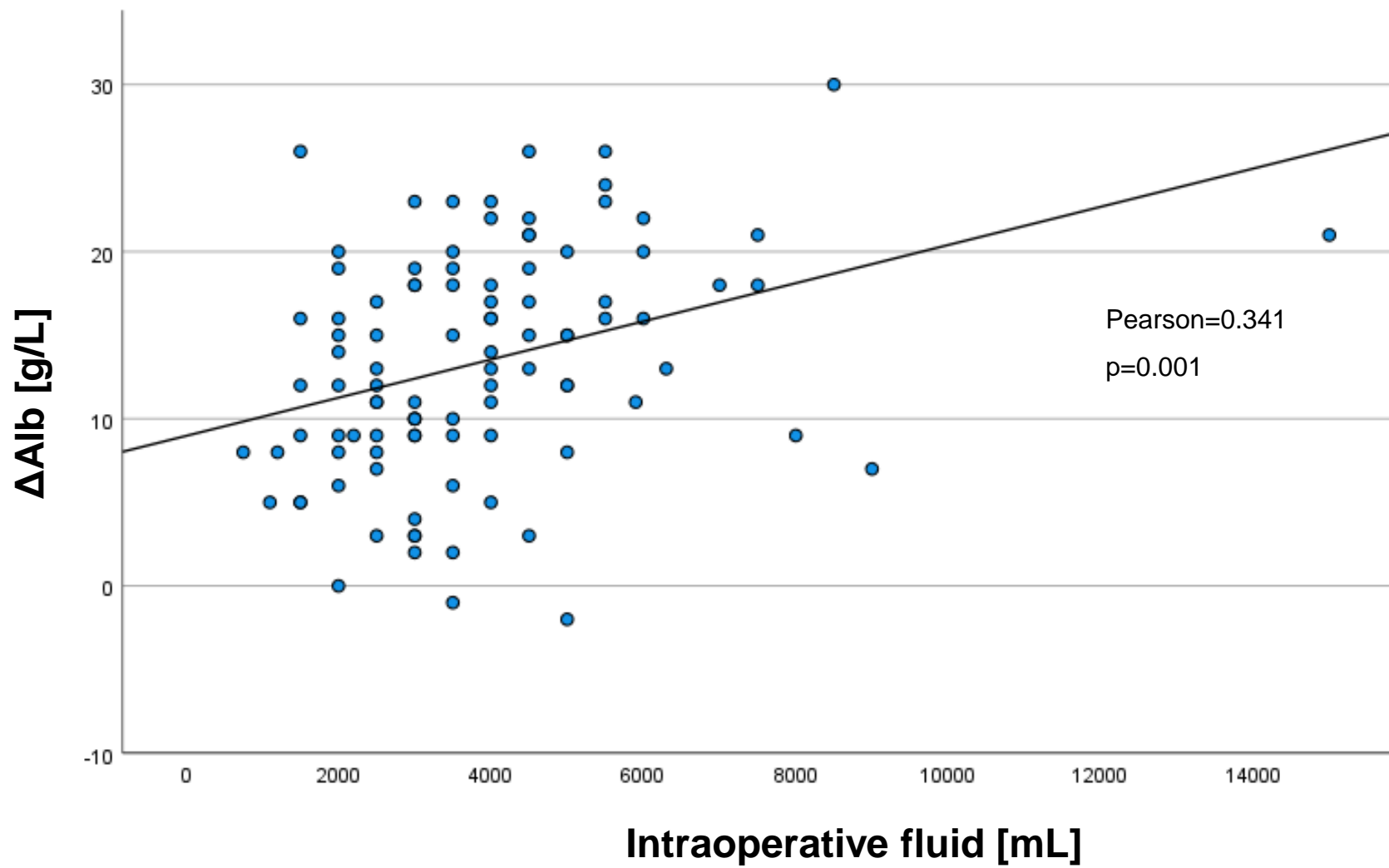

Supplement: Supplementary file 2 — Additional file 2: Supplementary Figure 1. Intraoperative fluid [mL]. [file 13741_2022_285_MOESM2_ESM.pdf]
